# Examining the use of different message categories to communicate AMR: a content analysis of instagram posts

**DOI:** 10.3389/fdgth.2025.1564584

**Published:** 2025-09-10

**Authors:** Sana Parveen, Wei-Jan Chang, Patricia McHugh, Akke Vellinga

**Affiliations:** ^1^ CARA Network, School of Public Health, Physiotherapy and Sports Science, University College Dublin, Dublin, Ireland; ^2^ J.E. Cairnes School of Business & Economics, University of Galway, Galway, Ireland

**Keywords:** AMR, public health communication, social media, public health messaging, public health campaigns, social marketing

## Abstract

**Background/objectives:**

Antimicrobial resistance (AMR) is a major worldwide concern with severe implications for public health, contributing to almost 5 million deaths in 2019. One of the main causes of AMR is overuse and misuse of antibiotics, which can be addressed by increasing awareness and educating the public on this issue. Studies have demonstrated the potential of social media to educate the public and influence behaviour. Instagram's unique features, such as its visual nature and user-friendly interface, make it well-suited for exploring health behaviours and disseminating information on various health topics. Research shows 65.4% of young adults (18–36 years old) use Instagram as their main source of information.

**Methods:**

This study examined AMR posts from Instagram from January 1, 2017 to July 15, 2023. In total, 4,606 images and videos were initially extracted which corresponded to 3,261 Instagram posts. After data cleaning, a final dataset of 574 posts were categorised into 6 message categories which were humour, shock/disgust/fear, personal stories/statements, educational/informative, opportunistic and advocacy.

**Results:**

The most common post category was educational/informative (78%) and humour was the least common (2%). We also looked at the average engagement (likes) with these posts, the educational/informative category received the most likes per post (mean of 30). The fear/shock/disgust category received 25 likes per post, humour and personal stories/statements 18 and 21 respectively.

**Conclusions:**

Our study shows Instagram has hardly been used for AMR interventions. An important population group of young adults who use Instagram as their main source of information, is missed in public health messaging on AMR.

## Introduction

When bacteria, viruses, fungi or parasites evolve and stop responding to medications that once worked to treat them this is known as antimicrobial resistance (AMR) (1). AMR is a major worldwide concern with severe implications for public health and healthcare systems (2). The inappropriate and excessive use of antimicrobials has given rise to the development and proliferation of resistant bacteria, leading to more difficult to treat infections, treatment failures and increased healthcare costs (3). In 2019, nearly 5 million deaths were attributed to AMR globally (2). To combat AMR and shape prescribing and consumption behaviour, effective communication about the use of antimicrobials and AMR is key (4).

### Need for AMR communication

Individuals’ knowledge, attitudes and beliefs about antimicrobials influence their usage (5). Social media use is ubiquitous and offers a powerful tool to raise awareness and offer potential for promoting behavioural change in health campaigns (6). However, its use and application is limited (4).

An AMR social media campaign in Sicily, Italy, used social media (Facebook, Instagram, Twitter and LinkedIn) and information technology which expanded the campaign's outreach and fostered behavioural change through monitoring, communication, promotion and targeted actions (7). Similarly, a campaign in South Africa successfully used Twitter to improve awareness of AMR, demonstrating its effectiveness in spreading messages (8).

YouTube has also been used to raise awareness about AMR through storytelling techniques. Qualitative analyses of the most-viewed YouTube videos identified a variety of storytelling genres and approaches, including personal anecdotes and fictional narratives, to convey the complexity of AMR (9).

### Use of social media in health messaging

Social media has become a rapid and effective means to communicate and share information. In recent years and in particular with the global COVID-19 pandemic, healthcare professionals use platforms such as X (formerly Twitter) and Instagram to connect with both their peers and the general public (10). Some interventions have used social media to spread information, engage with the target audience and encourage participation related to AMR (6).

X has emerged as a source of health-related information on the internet, given the wealth of information shared by both the public and official sources (11). It serves as a real-time global source of public health information and has been applied in public health research (12). X data has shown its use in various public health applications, including disease monitoring, gauging public sentiment, handling outbreaks and emergencies, making predictions, assessing lifestyle patterns and geolocation services (12).

### Use of Instagram in public health campaigns

In 2022, Instagram had more than 2 billion users of which the majority (61.2%) were between the ages 18–34 (13). It is estimated that individuals spend approximately 12 hours each month on Instagram, equivalent to about 30 min per day (14). Research shows 65.4% of young adults (18–36 years old) use Instagram as their main source of information (15).

Instagram's unique features, such as its visual nature and user-friendly interface, make it well-suited for exploring health behaviours and disseminating information on various health topics (16). Health influencers on Instagram share visually appealing content reaching millions of followers and effectively communicating health messages (17).

Organisations, such as government health agencies, are also leveraging Instagram to disseminate health information and engage with the public (18). A study categorised the top-ten health topics discussed on Instagram as acute illnesses, alternative medicine, chronic illnesses and pain, dietary practices, physical exercise, healthcare and medical information, mental well-being, musculoskeletal health and dermatology, sleep and substance use (19).

Research has shown that Instagram serves as an efficient means of communication, offering an alternative channel for health education and surveillance (20). This study aims to examine Instagram posts related to AMR, categorising them into specific message types and identifying which categories return the most public engagement.

In this research, we will explore the following questions:

1. What are the message types used in Instagram posts about AMR?
2. Which message categories generate the highest levels of public engagement in terms of likes? What is the frequency of these posts?

## Material and methods

### Data extraction

Data for this study was extracted from Instagram using the Apify web tool (Apify Instagram Scraper). Apify is an online web scrapping tool which helps in extracting data from the internet in an ethical manner, no personal information is extracted (21).

A search was conducted on Instagram to identify AMR-related content. The hashtags associated with these posts were examined to generate a preliminary list of potential keywords. This list was then reviewed and refined through discussion among the authors to finalise the keywords for extraction. Each finalised keyword was associated with a set of relevant hashtags to guide the data collection process (Supplementary Material S1). Keyword used to identify Instagram posts included:

• AMR
• Antimicrobial resistance
• Antibiotics
• Antimicrobials
• Antimicrobial stewardship
• Drug resistant
• Superbugs
• Infections
• Antibiotic prescribing
• Antibiotic resistance
• Bacterial infections

Data was extracted from January 1, 2017 to July 15, 2023. The data extracted for each post included post ID, URL, post type (image, video or carousel post), caption, hashtags, mentions, comments count, likes count, timestamp and owner ID.

In addition to textual data, images and videos were extracted from Instagram using a Python script (‘Instaloader’ package). In total, 4,606 images and videos were collected which accounted for 3,261 Instagram posts. Only publicly available data was extracted for this research and no identifiable or any personal information was used in the analysis.

### Data analysis

Data was cleaned and resulted in the removal of:

1. **Non-English Content**
2. **Irrelevant Content**: Posts that did not contain information related to AMR.
3. **Advertisements**: Any promotional or commercial content.
4. **Duplicate or Repeat Posts**: Multiple occurrences of the same content were deleted.

The final dataset included 896 images and 44 videos accounting for a total of 574 Instagram posts.

Posts were categorised into message categories to analyse the type of content, as the study is descriptive in nature. A deductive content analysis approach was used for categorisation (22). The message categories for this analysis were adapted from a previous study which examined COVID-19 post on X (23).

Posts were categorised according to the type of visual content and caption (messaging content) into 6 categories—Humour, Shock/Disgust/Fear, Personal stories/statements, Educational/Informative, Opportunistic and Advocacy. The coding process resulted in a dataset with each post assigned to one of the six message categories. This final categorized dataset was then used for further analysis and interpretation.

Two researchers (R1 and R2) coded 400 posts each, of which 200 posts were common. Additionally, researchers R3 and R4 coded 200 posts from the total 574 posts, of which 100 posts were common between them and the remaining posts overlapping with those coded by R1 and R2. The final categorisation was based on the most common category. Inter-rater reliability was based on the Kappa statistic which was calculated using R software (24). Cohen's kappa values ranged between 0.15 and 0.45 for the inter-coder agreement. The highest agreement was observed between R3 and R4 (*κ* = 0.45), while the lowest was between R1 and R4 (*κ* = 0.15). These findings show that coding consistency varied between pairs with the majority of values lying within the range of fair agreement (Supplementary Material S2).

Since the only engagement metric that could be extracted were likes, total engagement for each message category was calculated as the sum of likes across all posts in that category. For comparison across categories, the mean number of likes per post was also calculated for each message category.

## Results

**Figure 1:**
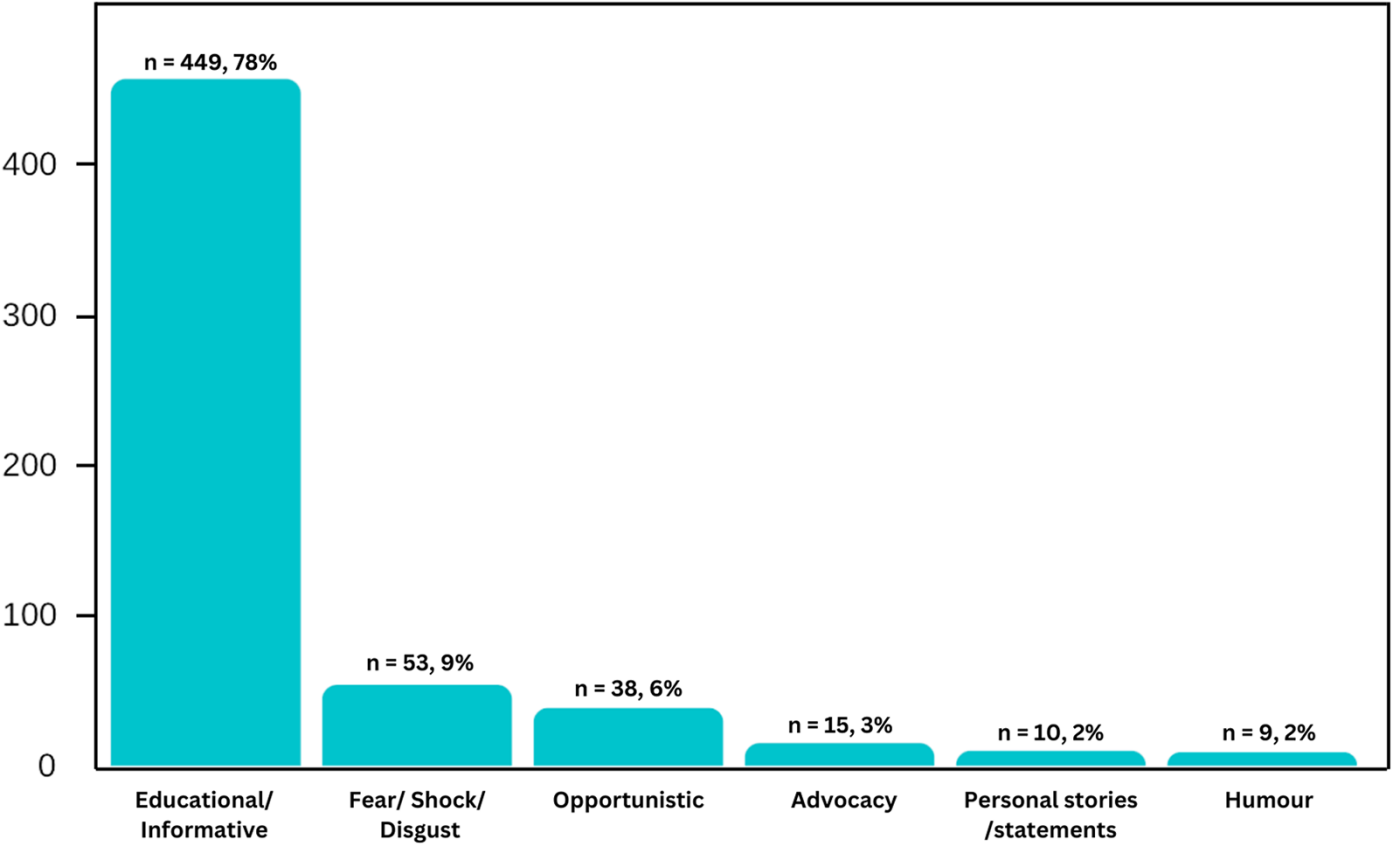
Classification of Instagram posts according to message type.

The final dataset included 574 posts over a period of 6.5 years. Figure 1 shows the classification of these posts according to message categories. The most common message category is Educational/ informative (78%), followed by Fear/Shock/Disgust (9%), Opportunistic (6%), Advocacy (3%), Personal stories/statements (2%) and Humour (2%).

The proportion of AMR messaging on Instagram increased over time from 9 posts in 2017 to 39 in 2018, 45 in 2020, 82 in 2021, 145 in 2022 and 225 in 2023 (6 months only) (Table 1).

**Table 1:** Frequency of messages on Instagram from 2017 to 2023.

Messages	2017	2018	2019	2020	2021	2022	2023 (6 months)
Advocacy	0	0	0	5	4	1	5
Educational/Informative	8	32	24	27	65	113	180
Opportunistic	0	2	0	3	6	10	17
Humour	0	0	2	0	2	1	4
Fear/shock/disgust	1	5	2	7	3	19	16
Personal stories/statements	0	0	1	3	2	1	3

The frequency of messages increased over time for most categories, in particular for Educational/Informative messages between 2021 (65 messages) and 2022 (113 messages) and 2023 (180 messages in the first 6 months). The Fear/Shock/Disgust category also increased while Advocacy, Opportunistic and humour category remained low.

The majority of AMR messaging on Instagram is focused on providing information about AMR and an increase in posts related to facts, debunking myths and antibiotic usage was observed during the World Antibiotics Awareness Week (WAAW) each year. For example, posts used messaging such as don’t take antibiotics for cold and flu, hand hygiene and not using leftover antibiotics frequently.

Engagement, measured in likes, with each of the message categories was highest for Educational/Informative (mean of 30), followed by Fear/Shock/Disgust (25 likes per post), Humour and Personal stories/statements (18 and 21 respectively), Advocacy (10) and Opportunistic (7) (Table 2).

**Table 2:** Total likes for each message category.

Messages	2017	2018	2019	2020	2021	2022	2023	Total likes	Mean like/post
Advocacy	0	0	0	119	22	3	13	157	10
Educational/informative	118	508	717	1,103	1,404	1,267	8,583	13,660	30
Opportunistic	0	38	0	68	37	39	96	278	7
Humour	0	0	18	0	62	58	22	160	18
Fear/shock/disgust	5	57	8	40	24	353	816	1,303	25
Personal stories/statements	0	0	66	54	12	71	9	212	21

## Discussion

The analysis and categorisation of posts allowed for a comprehensive exploration of Instagram posts related to AMR over a period of 6.5 years. Despite a quarter of the world population engaging with Instagram, this platform only provides a handful of posts (574) liked by just over 15,770 people/accounts.

Even though results indicate an increase in the volume of AMR-related Instagram posts over time, just 225 post were included for the first half of 2023. Most posts were Educational/Informative category, while Fear/Shock/Disgust posts also rose. Conversely, the Advocacy, Opportunistic, and Humour posts remained relatively low and stable.

In comparison, data pertaining to AMR on X with the identical set of 11 keywords, specifically focusing on the United Kingdom (UK) and Ireland resulted in a total of 49,726 tweets for the UK and 3,455 tweets for Ireland over the same period. This shows Instagram has not been utilised much for AMR campaigns missing an important target segment of young adults.

The included AMR Instagram posts were mainly Educational/Informative, showing its potential to educate. Most posts were related to facts, debunking myths and (reducing) antibiotic usage. Personal content has been shown to engage users and research shows that personalised content is most often shared on Instagram (25). However, few personal story posts were identified maybe explaining the lack of engagement. Future campaigns can include personalised content such as patient stories which could have more impact on the audience and potentially get more engagement as shown in previous research (26, 27).

The findings highlight the potential of educational content in AMR messaging on Instagram, with the most engagement for Educational/Informative posts. This engagement metric can guide future content creation to maximize reach and impact. Intervention planners can experiment with different types of educational content to determine which works best in raising awareness about AMR.

Previous studies have also highlighted the use of fear-based messaging to effectively communicate public health risks, which is consistent with the observed increase in Fear/Shock/Disgust posts (28, 29). The increase in Fear/Shock/Disgust posts and their engagement (25 likes per post) suggests that emotionally charged messages also resonate with the audience, though careful consideration is needed to balance fear appeals with constructive advice to avoid inducing undue anxiety or misinformation (30, 31).

The low engagement in Advocacy and Opportunistic categories may reflect a gap in leveraging these approaches for AMR awareness, suggesting potential areas for strategic enhancement. While Instagram is a prominent platform, there is a need for a creative approach to effectively engage diverse audiences, to ensure that AMR messages reach a broader audience.

Instagram seems to be underused for AMR messaging and education. As Instagram is a popular platform, in particular for younger people, the lack of its use is a missed opportunity. Influencers could potentially contribute to the spread of messages by liking or sharing AMR content (32, 33). Instagram offers a valuable channel for reaching a wide audience to address the global threat of AMR. Therefore, the use of Instagram in AMR messaging presents a promising avenue for public health campaigns aimed at raising awareness, educating the public and promoting responsible antibiotic use to combat antimicrobial resistance.

### Strengths and limitations

The main strength of this study is the comprehensive data collection over an extended period, providing a robust overview of trends in AMR-related content on Instagram. However, during data extraction, some posts could not be downloaded, which is most likely due to the fact that these posts originated from personal accounts, which are typically set to private. The exclusion of non-English language posts is a limitation. Also, using only likes to calculate engagement is another limitation of this study.

### Future research directions

Future research could explore the effectiveness of different message types in changing public attitudes and behaviours related to AMR. Additionally, investigating the role of influencers and targeted campaigns could provide insights to optimise AMR communication strategies on social media. A region-wise analysis could also be conducted to assess differences in engagement with AMR content across various geographical regions.

## Conclusion

When compared to other social media platforms, there is a notable lack of AMR messaging on Instagram. Health institutes, health professionals, and researchers should consider using the findings of this study as a starting point for their AMR campaigns on Instagram. Particularly, posting Educational/Informative, Fear/Shock/Disgust and Personal stories/statements messages to commence their campaigns. However, fear/shock/disgust messages need to be used with caution to avoid any negative impact of the campaign.

## Data Availability

The original contributions presented in the study are included in the article/Supplementary Material, further inquiries can be directed to the corresponding author.
